# Review on Neural Correlates of Emotion Regulation and Music: Implications for Emotion Dysregulation

**DOI:** 10.3389/fpsyg.2017.00501

**Published:** 2017-04-03

**Authors:** Jiancheng Hou, Bei Song, Andrew C. N. Chen, Changan Sun, Jiaxian Zhou, Haidong Zhu, Theodore P. Beauchaine

**Affiliations:** ^1^Center for Educational Neuroscience, School of Psychology and Cognitive Science, East China Normal UniversityShanghai, China; ^2^Department of Radiology, School of Medicine and Public Health, University of Wisconsin-MadisonMadison, WI, USA; ^3^Music Conservatory of HarbinHarbin, China; ^4^Center for Higher Brain Functions and Institute for Brain Disorders, Capital Medical UniversityBeijing, China; ^5^School of Education and Public Administration, Suzhou University of Science and TechnologySuzhou, China; ^6^Department of Psychology, Shihezi UniversityShihezi, China; ^7^Department of Psychology, The Ohio State UniversityColumbus, OH, USA

**Keywords:** music, emotion, emotion regulation, emotion dysregulation

## Abstract

Previous studies have examined the neural correlates of emotion regulation and the neural changes that are evoked by music exposure. However, the link between music and emotion regulation is poorly understood. The objectives of this review are to (1) synthesize what is known about the neural correlates of emotion regulation and music-evoked emotions, and (2) consider the possibility of therapeutic effects of music on emotion dysregulation. Music-evoked emotions can modulate activities in both cortical and subcortical systems, and across cortical-subcortical networks. Functions within these networks are integral to generation and regulation of emotions. Since dysfunction in these networks are observed in numerous psychiatric disorders, a better understanding of neural correlates of music exposure may lead to more systematic and effective use of music therapy in emotion dysregulation.

## Introduction

In the past two decades, considerable interest has emerged toward identifying neural substrates of emotion regulation (ER) (e.g., Davidson et al., 2000; Scherer and Zentner, 2001; Ochsner and Gross, 2005; Beauchaine et al., 2011; Campbell-Sills et al., 2011; Hilt et al., 2011; Cole et al., 2013; Beauchaine, 2015a,b). At the same time, interest in emotion-related neural processes evoked by music stimuli has also increased (Blood and Zatorre, 2001; Brown et al., 2004; Koelsch et al., 2006; Koelsch, 2010, 2014). Although it has long been known that exposure to music affects emotional experience and expression (Blood and Zatorre, 2001; Scherer and Zentner, 2001; Koelsch et al., 2013a,b), surprisingly little research has addressed the link between music and ER. This may be an important oversight, as effects of music on emotion regulatory processes may have therapeutic implications for a variety of clinical disorders, as we discuss below. Our major goal in writing this review is to explore what is known about the neural correlates of ER and music-evoked emotion, and to consider possible use of music therapy for emotion dysregulation.

## Emotion regulation

Emotion regulation (ER) includes the abilities to monitor, evaluate, and modify one's internal and external emotional reactions, and suppress aversive behavior toward others, in the service of adaptive functioning (e.g., Thompson, 1994; Gross and Thompson, 2007; Niven et al., 2009). ER invokes a set of complex processes across subjective experience (feeling states), cognitive responses (thoughts), autonomic responses (e.g., cardiac reactivity), and emotion-related behaviors (e.g., bodily actions, facial expressions). At times ER is automated, yet at other times it is effortful, requiring effective modulation of subcortically-mediated impulses by cortical brain regions (see Davidson, 2002; Heatherton, 2011; Heatherton and Wagner, 2011; Beauchaine, 2015b; Beauchaine et al., 2017).

Several volitional strategies to regulate emotions have been specified, including altering attention a situations, reinterpreting the meaning of situations (e.g., McRae et al., 2010), and change the nature of situations (e.g., Diamond and Aspinwall, 2003). Neural structures implicated in ER include frontal brain regions including the OFC, the ventrolateral PFC (vlPFC), and the dorsolateral PFC (dlPFC), depending on the emotion being regulated (for reviews see Gross and Thompson, 2007; Beauchaine, 2015b; Beauchaine et al., 2017). These frontal regions provide top-down inhibition of subcortical, limbic circuits that generate emotion, including the amygdala, hippocampus, insula, fusiform gyrus, and striatum, among others (Brendel et al., 2005; Kalisch, 2009; Dell'Osso et al., 2010; Holtmann et al., 2013; Sitaram et al., 2014). In fact, the amygdala (Kalisch, 2009; McRae et al., 2010), hippocampus (Phelps, 2003; Sripada et al., 2013), fusiform gyrus (Pollatos and Gramann, 2012; Fonville et al., 2014), striatum (Koelsch, 2014), and thalamus (Cheung et al., 2006) are all implicated in emotional reactivity, whereas the OFC (Ito, 2004; Rempel-Clower, 2007), vlPFC, dlPFC (Elliott, 2003; Chan et al., 2008; Levy and Wagner, 2011), and anterior insula (Sitaram et al., 2014) are implicated in effortful regulation of emotion (see Schulze et al., 2011).

## Music and emotion

Music has clear and measurable effects on emotional experience, and is almost universal in its emotional appeal (Scherer and Zentner, 2001; Peretz and Zatorre, 2005). Indeed, studies on neural correlates of music-evoked emotions have shown that music affects the functions implicated in emotion processing (i.e., amygdala, hippocampus, anterior congulate cortex, nucleus accumbens, and orbitofrontal cortex. See Blood and Zatorre, 2001; Peretz and Zatorre, 2005; Koelsch, 2010, 2014). Some researchers put forward that music may have an impact on emotion regulation (Koelsch, 2010, 2014; Moore, 2015) and may also improve the ability of emotion regulation when using music (especially with happy and pleasant feelings) as an intervention strategy on emotional regulation (Moore, 2013). Moore (2013) provided evidence that music evokes neural responding in the same regions that are implicated in effective ER. However, it should be noted that evoking emotions is not equivalent to ER; the former is passive whereas the latter usually involves goal-directed maintenance of appropriate emotional reactions. Thus, ER includes processes through which a person maintains behavior by modulating one or more aspects of emotion, with neural correlates involving an interplay among brain regions involved in emotion generation and executive control (Moore, 2013; Beauchaine et al., 2017). Below, we synthesize findings from studies that report on effects of music–evoked emotions, arranging our review across levels of neural functions, beginning with subcortical functions involved in emotion generation, and ending with cortical functions involved in ER (see Beauchaine, 2015b; Beauchaine and Zisner, in press).

### Subcortical emotion generation

#### Amygdala

Among other functions, the amygdala serves to initiate, integrate, maintain, and terminate emotions (Goldin et al., 2008; Pessoa, 2008). Early studies on amygdala indicated that it responds to diverse stimuli, including unpleasant music, that elicit negative feeling states (LeDoux, 1989; Gosselin et al., 2006; Koelsch et al., 2006; Lerner et al., 2009). Interestingly, such amygdala activation is decreased by both music improvisation (Limb and Braun, 2008) and pleasant music (Blood and Zatorre, 2001; Koelsch et al., 2006; Juslin and Västfjäll, 2008). Other studies demonstrate that the amygdala is also activated by positive stimuli (Pessoa et al., 2002; Hou, 2007), including music (Ball et al., 2007). Recent studies show that both the superficial and laterobasal subareas of amygdala are sensitive to pleasant and joyful music (Mueller et al., 2011; Koelsch, 2014). Furthermore, increased functional connectivity is observed between the superficial amygdala and the nucleus accumbens (NAcc), and between the superficial amygdala and the mediodorsal thalamus, when listening to joyful music compared to fear-evoking music (Koelsch et al., 2013a; Koelsch, 2014).

Especially, the laterobasal amygdala, which consists of the lateral, basolateral, basomedial, and paralaminar nuclei, is the amygdalar input structure for auditory and sensory information, and is involved in evaluation and learning of both positive and negative stimuli (Holland and Gallagher, 2004; Roozendaal et al., 2009). This region activates in response to both joyful and unpleasant music (Mitterschiffthaler et al., 2007; Koelsch, 2014), and appears to code the positive or negative reward value of assorted stimuli, including music (Koelsch, 2014). Notably, the laterobasal amygdala receives direct projections from the auditory cortex (LeDoux, 2000), and through such projections, the auditory cortex modulates laterobasal amygdala activity in response to complex sounds with emotional valence (Kumar et al., 2012). Thus, the amygdala shares interconnections with several integrative computational hubs, and exhibits increased functional activity and connectivity during emotional experience, including music-evoked emotion (Koelsch, 2014).

#### Hippocampus

The hippocampus is a limbic structure that plays important roles in spatial navigation and consolidation of information from short-term memory to long-term memory (Kesner and Dakis, 1995; Hartley et al., 2007). It is also involved in emotion (Phelps, 2004; Richter-Levin, 2004), including the responses to music with both positive (e.g., peaceful, joyous) and negative (e.g., unpleasant, sad) feeling states (Juslin and Västfjäll, 2008; Mueller et al., 2011; Trost et al., 2012). Functional connectivity between the hippocampus and the hypothalamus increases music-evoked joy, which supports the notion that the hippocampus is involved in music-evoked positive emotions, which in turn initiate neuroendocrine responses associated with reduced in emotional stress (Chanda and Levitin, 2013; Koelsch, 2014). Individuals with low capacity to experience positive emotions show decreased hippocampal volumes, and exhibit reduced neuronal activity in the hippocampus in response to musical stimuli (Koelsch et al., 2013a; Koelsch, 2014). Moreover, during the experience of negative music-evoked emotion, hippocampal activity may indicate automatic inhibitory processes to prevent hippocampal damage in response to acoustic stressors (Koelsch et al., 2006; Koelsch, 2014).

The laterobasal amygdala is implicated in regulating neural input to the hippocampus, and in inhibition (down-regulation) of hippocampal neural reactivity to unpleasant sounds (Roozendaal et al., 2009; Koelsch, 2010). Hippocampal damage can result in symptoms of depression and post-traumatic stress disorder (Koelsch, 2014). Drug and alcohol abuse can induce function damage to the hippocampus via reduced neurogenesis and loss of hippocampal neurons (Videbech and Ravnkilde, 2004; Warner-Schmidt and Duman, 2006). Moreover, lesions to the parahippocampal cortex and hippocampal subiculum result to music-evoked unpleasant emotions (Gosselin et al., 2006).

#### Nucleus accumbens (NAcc)

The NAcc, part of the ventral striatum, responds strongly to emotion (Floresco, 2015). It also responses to reward feeling such as the both primary and secondary reinforcers (e.g., food, water, money, sex) and pleasant music (Brown et al., 2004; Salimpoor et al., 2011; Trost et al., 2012). Studies show increased neural activity in the NAcc during music-evoked feelings of pleasure and reward (e.g., Blood and Zatorre, 2001; Menon and Levitin, 2005; Koelsch et al., 2006; Juslin and Västfjäll, 2008; Trost et al., 2012). Positron emission tomography (PET) studies indicate increased dopamine (DA) in the NAcc during exposure to pleasant music, which is probably released by mesencephalic DA neurons located in the ventral tegmental area (VTA; Menon and Levitin, 2005). A fMRI study showes the increased functional connectivity between the NAcc involved mesolimbic reward circuitry with released DA and other cortical regions (i.e., auditory cortices, ventromedial prefrontal regions) involved in sound perception, both of which then contributed to the esthetic rewards during music listening (Salimpoor et al., 2011). Thus, music-evoked pleasure is mainly associated with activation of the mesolimbic DA reward in NAcc pathway (see Koelsch, 2014).

Additional subcortical regions also respond to music-evoked emotions, including the insula, which may automatically regulate and represent/integrate emotionally relevant interoceptive sensory information, such as visceral reactions to music stimuli (Craig, 2009). Music that evokes positive emotions also elicits activity in the thalamus and putamen (the latter is part of the striatum; see Badgaiyan, 2010) via DA release (Pessoa et al., 2002; Trost et al., 2015). The thalamus is implicated in arousal and attentional processes (Paus, 2000), and its activation correlates with increased emotional saliency (Blood and Zatorre, 2001; Trost et al., 2015).

### Cortical emotion regulation

#### Anterior cingulate cortex (ACC)

The ACC is involved in decision-making, impulse control, and error-monitoring (Bechara, 2005; Noel et al., 2006; Boes et al., 2009), and in evaluating and modulating emotional reactions (Bush et al., 2000; Luu and Posner, 2003; Etkin et al., 2011). The ACC is activated by music stimuli that generate both pleasant (Blood and Zatorre, 2001) and sad (Berns and Moore, 2012) feeling states. ACC activation correlates with music modes (e.g., major vs. minor) and tempos (fast vs. slow) that convey happy vs. sad distinctions (Blood and Zatorre, 2001; Green et al., 2008; Koelsch, 2010; Lee et al., 2011; Moore, 2013). The left ACC is involved primarily in music rhythm- and melody-based working memory, though more strongly for rhythm (Jerde et al., 2011; Moore, 2013), and is activated when listening to favorable or energetic music (Brown et al., 2004). In contrast, the right ACC is activated during free generation tasks such as creative improvisation and pseudo-random performance (De Manzano and Ullén, 2012; Moore, 2013). There is increased activity in the ventral ACC compared to the dorsal ACC when listening to music (Green et al., 2008; Moore, 2013). Thus, ACC activation occurs when listening to music, when engaged in making music, and when attending to specific music characteristics (see Moore, 2013).

#### Orbitofrontal cortex (OFC)

The OFC is a cortical structure that is involved intricately in reward valuation and ER (Olson et al., 2007; Koelsch, 2010; Rouw et al., 2011; Beauchaine and Zisner, in press). Neuroimaging studies show music-induced increases in neural activity in OFC regions, and demonstrate that music can modulate orbitofrontal activity (Juslin and Västfjäll, 2008; Koelsch, 2010, 2014). Compared to the left OFC, the right OFC is activated during listening to negatively-valenced music and disliked songs (Berns and Moore, 2012). Some studies also demonstrate left OFC activity, whether participants listen to positive preferred music or unpleasant negative music (Blood and Zatorre, 2001; Flores-Gutierrez et al., 2007; Fujisawa and Cook, 2011). Of potential importance, music training can improve such OFC responses (Miendlarzewska and Trost, 2014), and gray matter loss/atrophy in the OFC is correlated positively with impairment of emotional responses to music (Omar et al., 2011; Koelsch, 2014). In addition, as above mentioned, the OFC and NAcc have an interactive effect on gaining reward during music listening (Salimpoor et al., 2011). Therefore, the OFC is responsible for emotion feeling as well as reward valuation.

#### Prefrontal cortex (PFC)

The PFC—a large brain region that covers most of the frontal lobes, is integral to effective ER (see e.g., Juslin and Västfjäll, 2008; Hilt et al., 2011; Beauchaine, 2015b; Beauchaine and Zisner, in press). The left PFC subserves positive emotional functions during listening to light, happy and joyful music, whereas the right PFC subserves negative emotional functions during aversive music presentations (Davidson et al., 2000; Eldar et al., 2007; Hou, 2007). Furthermore, the dorsolateral PFC (dlPFC) is founded to be activated during music improvisation (De Manzano and Ullén, 2012; Moore, 2013), and the ventrolateral PFC (vlPFC), located on the inferior frontal gyrus, is activated more during aversive music (Xiang et al., 2006; Hou, 2007). Another study confirmed this finding, and indicated that the vlPFC is activated when listening to dissonant music (Mutschler et al., 2010). In addition, vlPFC activation occurs during imagined singing, perhaps reflecting its role in emotional recall (Kleber et al., 2007; Moore, 2013).

### Cortical-subcortical networks of emotion regulation

Studies show that volitional regulation of emotion occurs through top-down cortical inhibition of subcortically generated affective states which is facilitated by cortical-subcortical connectivity (see e.g., Monk et al., 2008; Shannon et al., 2009; Pessoa and Adolphs, 2010; Beauchaine, 2015a). Music-evoked changes in amygdalar, hippocampal, NAcc, OFC, and PFC activation indicate that functions involved in emotion generation and regulation are affected by music stimuli. For example, during presentation of music, there is increased activation in the laterobasal amygdala, which shares functional interconnections with the hippocampus gyrus, whereas the superficial amygdala is connected functionally to the NAcc and OFC (Mitterschiffthaler et al., 2007; Koelsch, 2010).

The NAcc, ACC, insula, and hippocampus are all activated while participants listen to pleasant music (Blood and Zatorre, 2001; Brown et al., 2004). NAcc activation is observed concurrent with activation in the ventral tegmental area (VTA) and hypothalamus (Menon and Levitin, 2005). This suggests that hemodynamic changes observed in the NAcc reflect dopaminergic activity, since projections from the VTA to the NAcc form the dopaminergically-mediated “reward circuit” (Janata, 2009), including projections from the lateral hypothalamus to the VTA and NAcc (Koelsch, 2010).

The hippocampus shares dense reciprocal connections with structures involved in regulation of autonomic, hormonal, and immune system activity, including the amygdala, thalamus, hypothalamus, cingulate gyrus, and insula (Koelsch, 2010; McEwen and Gianaros, 2010). Thus, the hippocampus has major projections to both subcortical and cortical structures involved in emotional processing and its regulation (Koelsch, 2010).

## Potential use of music therapy for emotion dysregulation

In contrast to ER, the inability to modulate emotions in service of adaptive behavior is referred to as emotion dysregulation, which often has adverse consequences for mental health (Beauchaine, 2001; Saxena et al., 2011). In fact, emotion dysregulation characterizes most psychiatric disorders (Beauchaine, 2015a,b; Beauchaine and Thayer, 2015), including internalizing disorders (Mayberg et al., 2005; Cooney et al., 2010; Rajappa et al., 2012; Miranda et al., 2013), externalizing disorders (Beauchaine et al., 2007, 2017), autism (Ito, 2004; Neuhaus et al., 2014), post-traumatic stress disorder (PTSD; e.g., Falconer et al., 2008), obsessive-compulsive disorder (e.g., Milad and Rauch, 2012), and borderline personality disorder (BPD; e.g., Crowell et al., 2009; Sauder et al., 2016), among others (see Beauchaine, 2001; Beauchaine and Thayer, 2015). Individuals with BPD, for example, who suffer from poorly regulated impulsivity (see e.g., Derbidge and Beauchaine, 2014), exhibit frontolimbic dysfunction that includes striatal under-responding to incentives (Sauder et al., 2016), amygdalar hyperreactivity to emotion evocation (Bechara, 2005), and frontal hypoactivation during both types of tasks (Perez-Rodriguez et al., 2012; Sauder et al., 2016). Thus, limbic regions are not sufficiently modulated by the OFC and PFC—cortical substrates of effective ER (Crowell et al., 2009). Affected individuals also exhibit stronger functional connectivity between the amygdala and PFC during fear conditioning (Soloff et al., 2012). Finally, they exhibit structural brain abnormalities, such as reduced volumes in the OFC, dlPFC, amygdala, and hippocampus—all of which are implicated in ER (Kuhlmann et al., 2013; Niedtfeld et al., 2013; O'Neill et al., 2013; Krause-Utz et al., 2014). These findings suggest collectively that emotion dysregulation derives from ineffective modulation of subcortical emotion *generation* systems by cortical emotion *regulation* systems (Beauchaine, 2015b; see also Churchwell et al., 2009; Hilt et al., 2011). Although we discuss BPD as an illustrative example, similar findings apply to diverse forms of psychopathology across both the internalizing (depression, panic, anxiety) and externalizing (ADHD, CD, substance related disorders) spectra (see Monk et al., 2008; Shannon et al., 2009; Beauchaine and McNulty, 2013; Plichta and Scheres, 2014; Beauchaine, 2015b; Beauchaine and Thayer, 2015).

Evidence that music alters neural activity in these same functions and networks should encourage (1) more detailed investigations into the neural correlates of therapeutic effects of music, and (2) possible use of music in treatment of emotion dysregulation. At present, music has demonstrated the effects on depression through behavioral treatment, and is associated with reduced emotional reactivity in sadness-inducing contexts. For example, compared to depressed individuals who just receive standard care only, those who receive Active Music Therapy (AMT, the one which gives the person, the possibility to create his own acoustic with the help of musical instrument or with the voice. See Laura et al., 2015) plus standard care show greater improvement in depression and anxiety symptoms at 3-month follow-up (Erkkila et al., 2011). Neuroimaging studies show that listening to music can induce positive feelings and functional changes in the subcortical limbic structures as well as the frontal cortical regions that are implicated in depression and emotion dysregulation, music therapy therefore holds promise in helping depressed patients improve functioning of affect generating and affect regulating brain systems (Moore, 2013). By using the Music in Mood Regulation (MMR) therapy and fMRI, Carlson et al. (2015) found the responded activity of medial prefrontal cortex (mPFC) to the Discharge strategy (a method of MMR that makes negative feelings to be expressed. See Carlson et al., 2015), which reflected the improved scores on depression and anxiety. This neuroimaging study confirmes the active role of mPFC in the suppression of negative mood (Ochsner and Gross, 2005; Murakami et al., 2015) and also suggests that music can be applied with maladaptive patterns of emotion regulation (Carlson et al., 2015).

Therefore, given effects of music on the functions implicated in emotion regulation, music therapy, including strategic use of emotion-dampening music stimuli, might be beneficial for those with other chronic emotion dysregulation problems, such as individuals with BPD. As outlined above, those who are affected by BPD show impaired amygdalar, hippocampal, ACC, OFC, and dlPFC function (Nunes et al., 2009; Pessoa et al., 2012; Soloff et al., 2012; Niedtfeld et al., 2013), with resulting ER difficulties. Although certain behavioral methods help to treat chronic emotion dysregulation observed in BPD (see Linehan, 1993; Lynch et al., 2007; Ritschel et al., 2012), such methods primarily involve re-interpretation of external cues such as facial expressions and body movements, from which rapid assumptions about others' underlying thoughts and feelings states are gleaned (Bateman and Krawitz, 2013). More recently, focus has shifted to interoceptive exposure in which internal emotional states (e.g., anxiety) are altered toward changing negative emotions (e.g., Otto et al., 2005; Baker et al., 2007). Through music therapy, clients improve their affective states and behaviors by using music experiences such as free improvisation, singing, listening to, discussing, and moving to music to achieve treatment goals that improve ER (Otto et al., 2005). Music therapy may also result in reduced quantity of pharmacological agents administered, or even replace medication in some cases (Tournaire and Theau-Yonneau, 2007; Lindsey, 2009). Thus far, however, almost no studies have explored direct effects of music exposure on emotion dysregulation, so feasibility has yet to be demonstrated.

## Future directions

Future studies are needed to systematically investigate the efficacy of music therapy among those with ER difficulties, and to evaluate effects of such therapy on neural functions and networks that subserve core ER. Thus, there is a need for additional research, especially given how important adaptive ER skills are for mental health, responses to stress, and the capacity to develop healthy relationships. Given that music may naturally facilitate ER (Moore, 2013), music-based treatment may enhance adaptive neuro-developmental responses by providing real-time interactive opportunities to manage emotionally evocative experiences (Moore, 2015). Future research will tell.

## Author contributions

JH: put forward the review idea, read lietrature, organize article structure, write and revise manuscript; BS: read lietrature, organize article structure, help revise manuscript; ACN: help organize article structure, revise manuscript based on his research experience (mental and brain disorder); CS: revise manuscript based on his research experience (emotion and brain function); JZ: revise manuscript based on her research experience (emotion education and neural basis); HZ: provide the reference to article structure, help revise on music and emotion section; TB: help revise the manuscript on article structure, logical organization and language (to be more professional and native).

## Funding

This research is sponsored by: (1) Peak Discipline Construction Project of Education and Large Instruments Open Foundation at East China Normal University; (2) The 58th China Postdoctoral Science Foundation Funded Project (No. 2015M580303); Beijing Institute for Brain Disorders (BIBD-PXM2013_014226_07_000084).

### Conflict of interest statement

The authors declare that the research was conducted in the absence of any commercial or financial relationships that could be construed as a potential conflict of interest.
